# Surface modification of Polyether-ether-ketone for enhanced cell response: a chemical etching approach

**DOI:** 10.3389/fbioe.2023.1202499

**Published:** 2023-09-07

**Authors:** Rupak Dua, Onessa Sharufa, Joi Terry, William Dunn, Indu Khurana, Jagasivamani Vadivel, Yue Zhang, Henry J. Donahue

**Affiliations:** ^1^ American Dental Association Science and Research Institute (ADASRI), Gaithersburg, MD, United States; ^2^ Department of Chemical Engineering, Hampton University, Hampton, VA, United States; ^3^ Department of Biology, Hampton University, Hampton, VA, United States; ^4^ The New Horizons Governor’s School for Science and Technology, Hampton, VA, United States; ^5^ Department of Economics and Business, Hampden-Sydney College, Hampden-Sydney, VA, United States; ^6^ Department of Biomedical Engineering, Virginia Commonwealth University, Richmond, VA, United States

**Keywords:** biomaterials, PEEK-Polyether-ether-ketone, dental implant, orthopedic implant, chemical etching

## Abstract

Polyether-ether-ketone (PEEK) is increasingly becoming popular in medicine because of its excellent mechanical strength, dimensional stability, and chemical resistance properties. However, PEEK being bioinert, has weak bone osseointegration properties, limiting its clinical applications. In this study, a porous PEEK structure was developed using a chemical etching method with 98 wt% sulfuric acids and three post-treatments were performed to improve bone cell adhesion and proliferation. Four groups of PEEK samples were prepared for the study: Control (untreated; Group 1); Etched with sulfuric acid and washed with distilled water (Group 2); Etched with sulfuric acid and washed with acetone and distilled water (Group 3); and Etched with sulfuric acid and washed with 4 wt% sodium hydroxide and distilled water (Group 4). Surface characterization of the different groups was evaluated for surface topology, porosity, roughness, and wettability using various techniques, including scanning electron microscopy, profilometer, and goniometer. Further chemical characterization was done using Energy-dispersive X-ray spectroscopy to analyze the elements on the surface of each group. Bone cell studies were conducted using cell toxicity and alkaline phosphatase activity (ALP) assays. The SEM analysis of the different groups revealed porous structures in the treatment groups, while the control group showed a flat topology. There was no statistically significant difference between the pore size within the treated groups. This was further confirmed by the roughness values measured with the profilometer. We found a statistically significant increase in the roughness from 7.22 × 10^−3^ μm for the control group to the roughness range of 0.1 µm for the treated groups (Groups 2–4). EDX analysis revealed the presence of a 0.1% weight concentration of sodium on the surface of Group 4, while sulfur weight percentage concentration was 1.1%, 0.1%, and 1.4% in groups 2, 3, and 4, respectively, indicating different surface chemistry on the surface due to different post-treatments. Cell toxicity decreased, and ALP activity increased in groups 3 and 4 over 7 days compared with the control group. It is demonstrated that the surface modification of PEEK using a chemical etching method with post-processing with either acetone or sodium hydroxide provides a nano-porous structure with improved properties, leading to enhanced osteoblastic cell differentiation and osteogenic potential.

## 1 Introduction

Most of the implants used in orthopedic and dental applications are composed of metallic alloys including titanium alloys, stainless steel, and cobalt-chromium (Litak et al., 2022; Xing et al., 2022). Corrosion and wear debris caused by these metallic implants affect the surface and biocompatible behavior that induces tissue reactions which might lead to the release of corrosive byproducts and ions from the implant surface resulting in premature failure of implants (Messous et al., 2021; Amirtharaj Mosas et al., 2022; Matsumae et al., 2022). In addition, it is essential to note that load-bearing joint metallic implants due to their high stiffness, do not effectively distribute the load to the surrounding bone. This phenomenon is known as stress shielding and can result in bone remodeling and increased bone resorption. Over time, this can lead to implant loosening, requiring extensive and expensive revision surgeries (Kanayama et al., 2000; Mi et al., 2007; Gok, 2022). Besides, postoperative assessment of bone growth around the metallic implant or examining other issues around the implant is challenging using traditional imaging procedures, like MRI or CT, due to the metal implant’s radiopacity (Cizek and Boyd, 2000). As a result, there has been a growing search for an alternative material that can overcome the challenges presented by metallic implants. Polyether-ether-ketone (PEEK) polymer is increasingly being used in many fields, including biomedical applications such as orthopedic and dental implants (Kurtz and Devine, 2007; Dua et al., 2021). PEEK is a polyatomic semi-crystalline aromatic thermoplastic polymer that is biocompatible, chemically inert, high-temperature resistant, and has mechanical properties resembling human bone (Harsha and Tewari, 2003; Toth et al., 2006; Panayotov et al., 2016; Tekin et al., 2018). Further, PEEK can also be additively manufactured into complex forms (Haleem and Javaid, 2019; Zhang et al., 2019) and is translucent to X-rays, which allows the use of conventional radiographic imaging (Johansson et al., 2018).

However, PEEK is bioinert and has weak bone osseointegration properties that hamper its complete usage in orthopedic and dental applications (Chen et al., 2017). This implies that when PEEK is employed in medical implants or devices that interact with bone, it fails to facilitate robust attachment or integration with the neighboring bone tissue. Consequently, the clinical utility of PEEK is constrained, hindering its widespread application in the medical field. It has been found that the surface texture of a material plays a crucial role in regulating the behavior of adherent cells (Alves et al., 2010; Chen et al., 2018; Elliott et al., 2021). Modifying the surface texture at the nanoscale appropriately may enhance the adherence and proliferation of cells and promote tissue integration into the PEEK implant. Chemical etching of PEEK offers an attractive method to modify the surface of PEEK cost-effectively. There have been previous studies that have performed post-polymer sulfonating of PEEK with different concentrations of sulfuric acid (ranging from 95–98 wt%) (Huang et al., 2001; Wang et al., 2019), with varying durations of time (ranging from 5 s to greater than 30 min) (Zhao et al., 2013; Wang et al., 2019) and post treatments (Shukla et al., 2012; Wang et al., 2019) to create a porous structure on PEEK to improve osteogenic behavior. However, with so many different protocols with the difference in sulfuric acid concentration, duration of treatment and post-treatments, it was unclear which treatment was better to promote bone cell adhesion and osteoblastic differentiability.

So in this study, we attempted to modify the surface of PEEK at the nanoscale systematically using different post-processing chemical methods with fast ambient room temperature sulfonation that was previously optimized for the post-treatment of sodium hydroxide only using 98wt% sulfuric acid (Wang et al., 2019). We hypothesize that the various nanostructures created through different treatment methods will produce and elicit distinct surface chemistry, impacting the cytotoxicity and differentiation of osteoblastic cells when exposed to PEEK surfaces.

## 2 Materials and methods

### 2.1 Preparation of polyether-ether-ketone samples

PEEK sheets with a thin plastic protective cover with dimensions 6″ X 6″ X 1/16″ were bought from the supplier (McMaster-Carr Elmhurst, IL). The sheet was cut into smaller 0.7 × 0.7 mm sections using a band saw, keeping the height same as the sheet. The sides of the sections were sanded to remove rough surfaces generated using the band saw. The plastic protective covering on each smaller section was then removed before performing any surface treatment on the PEEK samples.

### 2.2 Chemical etching treatment

The surface of PEEK samples was treated with 98 wt% sulfuric acid (Sigma-Aldrich Inc., St. Louis, MO) for 30 s at ambient room temperature to modify the surface at the nanoscale. The high concentration of sulfuric acid and short time duration was chosen to achieve fast sulfonation while still causing chemical etching, as previously found in the literature (Wang et al., 2019). After chemically etching the samples, each treated sample was immersed in deionized (DI) water and manually moved slowly in a smooth circular motion using forceps for 20 s to remove the residual sulfuric acid on the surface. After immersion in DI-water, three post-treatments were performed to generate different surface chemistries on the treated groups. Treatment 1: After immersion in DI water, each sample was further rinsed in DI water for 1 minute ultrasonically at a frequency of 40 KHz three times. Treatment 2: After swirling samples in DI water, the samples were immersed in acetone for 30 s before rinsing with DI water ultrasonically three times. Treatment 3: After swirling samples in DI water, the samples were immersed in 4 wt% sodium hydroxide (Sigma-Aldrich Inc.) solution for 30 s before rinsing with DI water ultrasonically three times. All the PEEK samples from each treatment group were air-dried before using them for subsequent experiments.

In sum, four (4) groups of PEEK samples were used in our study. Group 1: PEEK samples without any treatment acted as a control group. Group 2: PEEK samples that underwent Treatment 1 (30s Sulfuric Acid +20 s DI water immersion + DI Water Wash). Group 3: PEEK samples that underwent Treatment 2 (30 s Sulfuric Acid +20 s DI water immersion +30 s Acetone immersion + DI Water Wash). Group 4: Samples that underwent Treatment 3 (30 s Sulfuric Acid +20 s DI water immersion +30 s NaOH immersion + DI Water Wash). In the alkaline phosphatase activity experiment, we also used tissue culture polystyrene (TCPS) as a positive control in which cells were cultured on a well of a 24-well plate.

### 2.3 Surface morphology and porosity using scanning electron microscopy and energy dispersive X-Ray spectroscopy

PEEK discs from all groups were observed under the scanning electron microscope to evaluate the unique structures developed on the surface due to chemical etching and post-treatment methods. Samples from each group (n = 3) were first sputter coated with gold to a thickness of 10 nm using a Luxor Gold Coater (Luxor Tech, Nazareth, Belgium) before they were examined under a desktop SEM (Phenom Pure G6, NanoScience Instruments, Phoenix, AZ) operating under 10 kV. Images were captured with a magnification of ×20,000 and 400,00x and were analyzed for surface topology. Further, multiple pores were randomly selected in the representative images of each group, and their diameter was measured using PhenomImageViewer software to determine the porosity of the surface. EDX measurements were also conducted to assess the elemental composition on the surface of the PEEK samples for each group.

### 2.4 Wettability

Contact angles were quantified using the sessile drop method with an Ossila Contact Angle Goniometer (Ossila Limited, Sheffield, United Kingdom), employing DI water as a solvent, as previously described in our earlier study (Gill et al., 2015). A drop of water was placed on the surface of each group of samples (n = 3/group), and images were recorded on a high-resolution camera. Multiple tests were conducted under ambient conditions at various locations on each sample with sufficient spacing to prevent any interference from previous tests. Contact angle measurement software that came with the instrument was employed to evaluate the wettability properties of the surface in terms of the contact angle θ, according to Young’s Equation (ϒsv = ϒls + ϒlv · cosθ), where ϒsv, ϒls, and ϒlv are the forces exerted by the surface tensions at three interfaces: solid-vapor, liquid-solid, and liquid-vapor respectively. The contact angle θ is the angle between a solid surface and the liquid interface.

### 2.5 Roughness

The roughness of the PEEK surface for each group (n = 3/group) was analyzed using an Alpha Step D-300 Stylus Profiler (KLA-Tencor Corporation, Milpitas, CA). The stylus of the profiler was moved a 3 mm distance three times on different areas of the samples to provide a raw roughness value. Raw roughness values were passed through a high-pass filter to separate the roughness from waviness. Average values for filtered roughness for each group were recorded and compared.

### 2.6 Osteoblast culture and studies

Human bone-forming pre-osteoblastic cells (hFOB 1.19, CRL-11372 ATCC, Manassas, VA) employed in our prior studies were selected as the cellular model for assessing the toxicity associated with different surface groups (Lahiri et al., 2012; Dua et al., 2014). The effect of the different surfaces on osteoblastic differentiation was examined by assessing the alkaline phosphatase activity. The PEEK samples from various groups were sterilized by spraying them with a 70% ethanol solution, followed by air drying. Subsequently, the dried samples were exposed to UV radiation with a wavelength of 254 nm for 30 min. This was done before conducting any cell culture studies on the samples to ensure an optimal sterile environment.

#### 2.6.1 Culturing of osteoblastic cells

Osteoblastic hFOB 1.19 cells were cultured in a complete growth medium as recommended by the manufacturer. Briefly, frozen cells were thawed and transferred into media comprised (Catalog # 11039021, Gibco, Fischer Scientific, Hanover Park, IL), which is a 1:1 mixture of DMEM/F-12 with 2.5 mM L-glutamine supplemented with 10% fetal bovine serum (Catalog # 16000044, Fischer Scientific), 1% penicillin/streptomycin (Catalog# 15140122, Fischer Scientific) and 0.3 mg/ml of G418 disulphate solution (Catalog# G8168, Sigma Aldrich Inc.) Upon arrival to our laboratory, the cells were culture expanded as previously reported (Dua et al., 2014) until passage 4 (P4) in a humidified incubator at 34°C and 5% CO_2_ as recommended by the manufacturer. Culturing osteoblasts at 34°C helps to replicate the *in vivo* conditions and mimic the microenvironment in bone tissue more accurately than at higher temperatures, such as 37°C, which is the standard for most cell culture experiments, allowing for more reliable and relevant experimental results. Additionally, culturing osteoblasts around 34°C has been observed to enhance their differentiation and functionality. It promotes the expression of bone-specific genes and the production of extracellular matrix components, thereby better simulating the natural bone formation process (Harris et al., 1995; Subramaniam et al., 2002).

#### 2.6.2 Cytotoxicity evaluation

Osteoblastic hFOB 1.19 cells were seeded on all surface groups (n = 3 surface samples/group) at a cell density of 5.2 ×10^4^ cells/cm^2^ in a 24-well plate. The plates were stored in a standard cell culture incubator (humidified environment at 5% CO_2_ and 34°C) with a complete media change after every 3 days. Subsequently, sulforhodamine B (SRB) assays were performed as previously reported (Lahiri et al., 2012) on day 1, day 3, and day 7 following incubation to assess cell viability using CytoScan™ SRB Cytotoxicity Assay Kit (Catalog # 89028079, Fischer Scientific). The SRB assay is based on colorimetric measurement of viable cellular protein. In brief, the cells were first affixed onto the PEEK samples using a fixative agent, trichloroacetic acid (TCA), and incubated for 1 h at 4°C. Next, the samples were washed 3–4 times with water to remove excess fixative. The samples were then air-dried overnight before staining with the SRB dye solution for 30 min. Finally, the unbound dye was removed using a dye wash solution. The SRB dye bound to cellular protein was extracted using SRB solubilization buffer, and absorbance was measured (565 nm wavelength) using a microplate reader (Synergy HT, Biotek Instruments, Inc., Winooski, Vermont). Cellular viability was reported based on “Relative Survival” which was determined from the absorbance values measured (n = 3 samples/group). A normalization process was carried out for reporting purposes like previous studies (Lahiri et al., 2012; Dua and Ramaswamy, 2013). Results were normalized such that the average absorbance of the control (Group 1) was equal to one. Specifically, the background absorbance was initially subtracted from each of the individual absorbance values. Next, normalization was performed by dividing each of the absorbances (average and standard deviation values) by the average absorbances of the corresponding control group of cells. Data for each group was presented as mean ± standard error (SE). A higher OD value reflects decreased cell toxicity due to more number of cells present.

#### 2.6.3 Alkaline phosphatase activity

Osteoblastic differentiation was assessed by measuring alkaline phosphatase (ALP) activity via a fluorometric assay (Catalog # 89028079, Fischer Scientific). Cells were seeded on the samples for each group (n = 3/group) within 24-well plates at a density of 5.2 ×10^4^ cells/cm^2^. Cells from the samples for each time point were removed using trypsin and homogenized in 100 µL of assay buffer. 30 μL samples were taken in replicates of 3 for each sample in 96-well plates. Next, 80 µL of assay buffer was added to make a net volume of 110 µL in the wells. In addition, background samples were made by taking 30 µL of test samples and adding 80 µL of assay buffer and 20 µL of stop solution. After that, 20 µL of 0.5 mM of non-fluorescent 4 Methylumbelliferyl phosphate disodium salt (MUP) substrate was added to each well containing the test and background samples. The samples were then covered to prevent exposure to light, and the reaction was held at 25°C for 2 h. Next, the reaction was halted by the addition of 20 µL stop solution to each well (except for the background samples) and gently shaken. The fluorescence intensity was measured as per the manufacturer’s instructions using a fluorescence microplate reader at Ex/Em 360/400 nm. Values for the test samples were corrected by subtracting the value derived from the sample background controls. The amount of 4-MU generated was calculated from the standard curve that was also made as per the manufacturer’s instructions. ALP activity was calculated in mU/ml using the formula ALP activity = A/V/T where.

A = Amount of 4-MU generated by samples (in nmol)

V = Volume of the samples added in the assay well (in ml)

T = Reaction time (in minutes)

Data for ALP for each group was presented as mean ± standard error (SE).

### 2.7 Statistics

All data are expressed as mean ± standard deviation (SD) if otherwise stated. Statistical analyses of the results obtained for the wettability and roughness were performed using commercially available software (SPSS, IBM, version 27, Armonk, NY). A one-way ANOVA and *post hoc* Tukey test were used to compare means and determine statistically significant differences (*p* < 0.05) between groups.

## 3 Results

**FIGURE 1:**
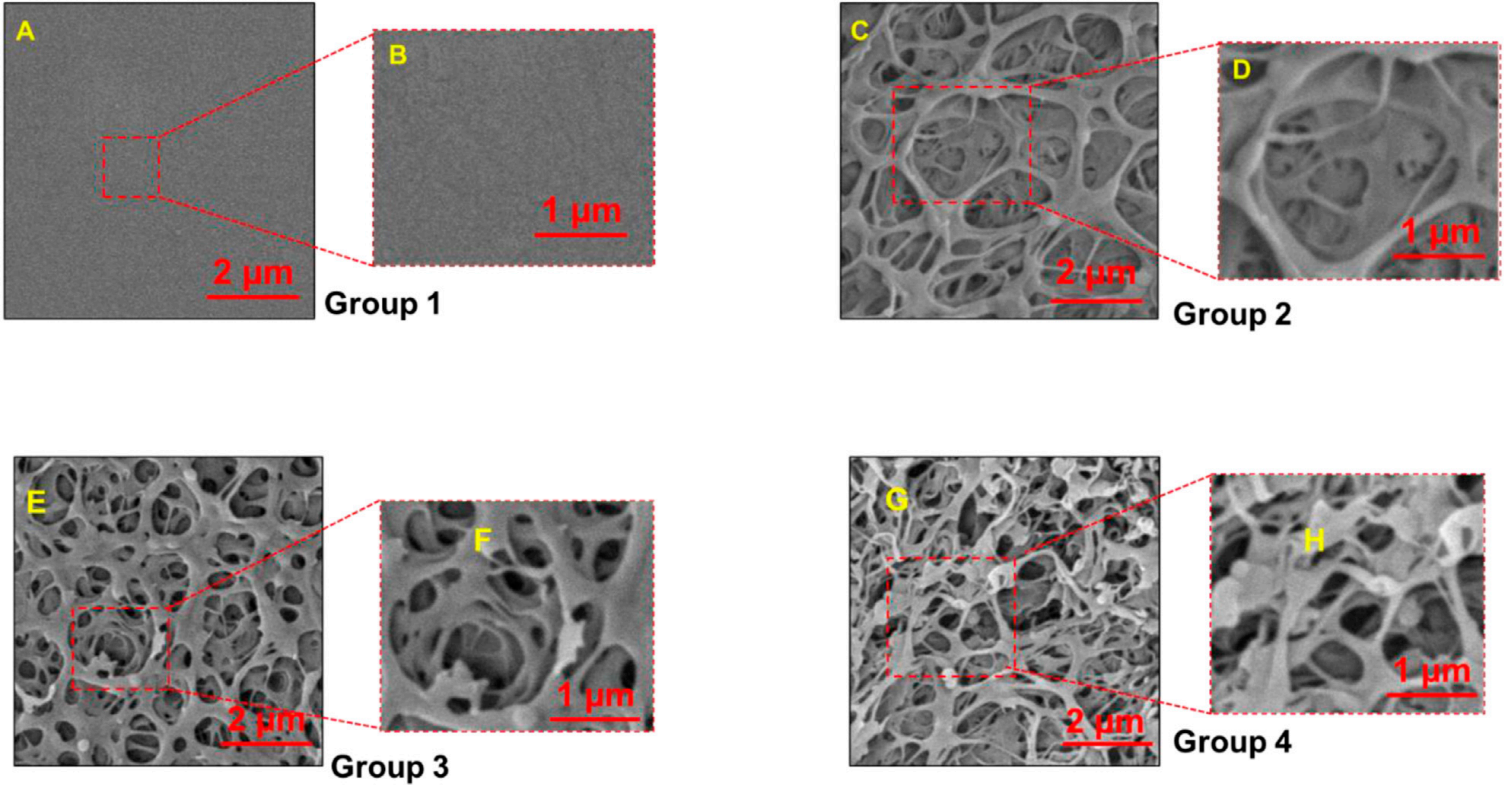
Scanning Electron Microscopy (SEM) images of PEEK surfaces magnified to ×20,000 **(A,C,E, G)** and 400,00X **(B,D,F, H)**. Group 1—Control; Group 2—Treatment 1 (30s Sulfuric Acid +20s DI water immersion + DI Water Wash); Group 3—Treatment 2 (30s Sulfuric Acid +20s DI water immersion +30s Acetone immersion + DI Water Wash); Group 4 - Treatment 3 (30s Sulfuric Acid +20s DI water immersion +30s NaOH immersion + DI Water Wash). Group 1 exhibited flat smoot surface while pores were observed in Group 2, 3 and 4.

n our investigation, we have observed distinct surface topologies among the various PEEK groups, as visually depicted in Figure 1. The control group, serving as our baseline, exhibited a remarkably smooth and even texture devoid of any discernible pores.

### 3.1 Surface morphology and porosity

We found different topologies on the surface of the various PEEK groups as depicted in Figure 1. The control group, serving as our baseline, exhibited a remarkably smooth and even texture devoid of any discernible pores. The control group had a very smooth and flat texture without any pores present. In contrast, the treatment groups (Groups 2, 3, and 4) displayed intricate porous nanostructures that exhibited pronounced variations in terms of depth and distribution within the material.

Next, pore sizes were measured using the PhenomImageViewer. The average pore sizes measured by randomly selecting nine pores on a sample image (n = 3/group) were 1.24 ± 0.29 µm, 1.02 ± 0.28 µm, and 1.46 ± 0.33 µm for groups 2, 3, and 4, respectively. We found no statistically significant difference in the pore size measured for treatment groups, except when compared to the control samples.

### 3.2 Energy dispersive X-Ray spectroscopy

From EDX analysis, we found that no sulfur and sodium were present on the control surface, while there was the presence of a 0.1 weight % concentration of sodium on the surface of Group 4 only. Sulfur percentage weight concentration was 1.1, 0.1, and 1.4 in groups 2, 3, and 4, respectively (Table 1). We also observed an increase in the oxygen weight % and a decrease in the carbon weight % for the treatment groups compared with the control samples.

**TABLE 1:** Weight % concentration of different elements in the PEEK samples for four groups using EDX.

Groups	Elemental analysis (weight %)				
	Carbon (C)	Nitrogen (N)	Oxygen (O)	Sulphur (S)	Sodium (Na)
Group 1	74.6	4.5	20.9	-	-
Group 2	74.0	3.6	21.3	1.1	-
Group 3	74.0	4.7	21.2	0.1	-
Group 4	71.4	4.4	22.7	1.4	0.1

Group 1—Control; Group 2—Treatment 1 (30 s Sulfuric Acid +20 s DI water immersion + Water Wash); Group 3—Treatment 2 (30 s Sulfuric Acid +20 s DI water immersion +30 s Acetone immersion + Water Wash); Group 4—Treatment 3 (30 s Sulfuric Acid +20 s DI water immersion +30 s NaOH immersion + Water Wash)

### 3.3 Wettability

**FIGURE 2:**
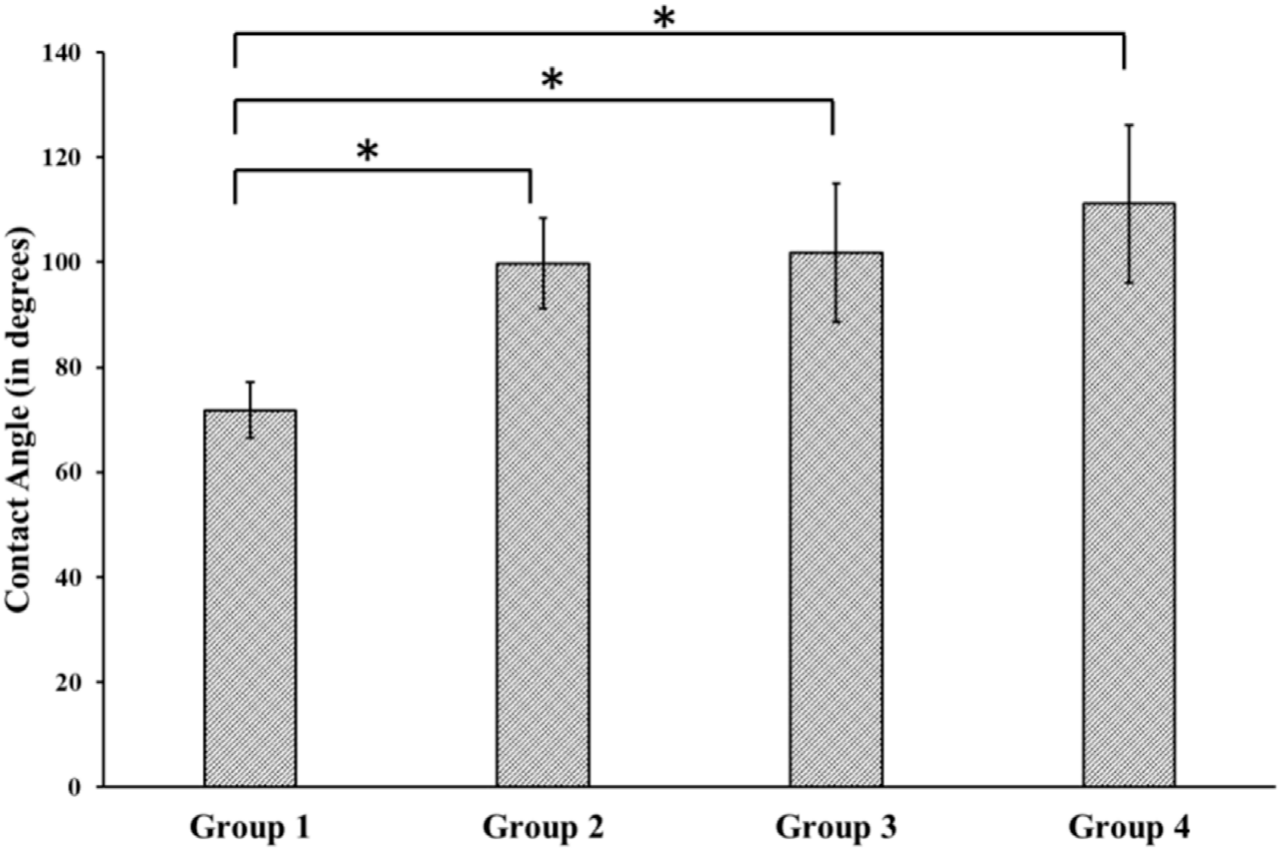
Contact Angle measurement using Goniometer for different groups. Group 1—Control; Group 2—Treatment 1 (30s Sulfuric Acid +20s DI water immersion + DI Water Wash); Group 3—Treatment 2 (30s Sulfuric Acid +20s DI water immersion +30s Acetone immersion + DI Water Wash); Group 4 - Treatment 3 (30s Sulfuric Acid +20s DI water immersion +30s NaOH immersion + DI Water Wash). The “*” indicates that the difference between the groups was statistically significant (*p* < 0.05).

The average contact angle measured for Group 1 (control) surface was 71.85 ± 5.31^o^. However, there was a statistically significant increase in the contact angle for the treated group when compared with the control group. The average contact angle measurement was found to be 99.78 ± 8.61^o^, 101.87 ± 13.17^o^ and 111.15 ± 15.04^o^ for groups 2, 3, and 4, respectively, but the differences between the contact angles for the 3 treatment groups were not statistically significant (Figure 2).

#### 3.4 Roughness

**FIGURE 3:**
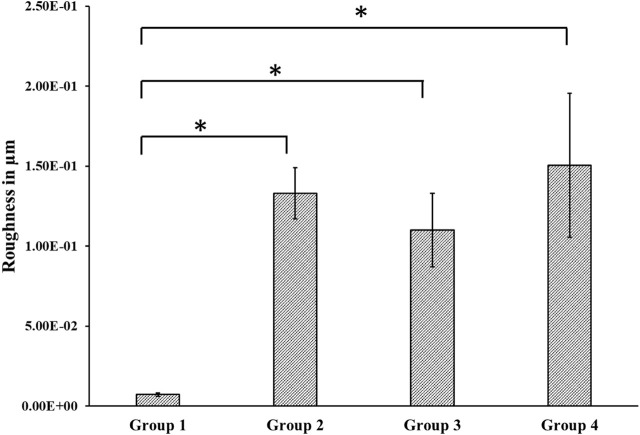
Roughness measured using Alpha Step D-300 Stylus Profiler. We found a statistically significant difference between roughness values of the control and the treated groups. Group 1—Control; Group 2—Treatment 1 (30s Sulfuric Acid +20s DI water immersion + DI Water Wash); Group 3—Treatment 2 (30s Sulfuric Acid +20s DI water immersion +30s Acetone immersion + DI Water Wash); Group 4 - Treatment 3 (30s Sulfuric Acid +20s DI water immersion +30s NaOH immersion + DI Water Wash). The “*” indicates that the difference between the groups was statistically significant (*p* < 0.05).

We found a relatively smooth surface for the control group with a roughness value of 7.22 × 10^−3^ ± 0.001 µm, which was statistically significantly different from the treatment groups’ roughness values (Groups 2–4). The average roughness values were 0.13 ± 0.02 µm, 0.11 ± 0.02 µm, and 0.15 ± 0.04 µm for groups 2, 3, and 4, respectively. However, there was no statistically significant difference in the roughness values between the treated groups (Figure 3).

#### 3.5 Cytotoxic evaluation

**FIGURE 4:**
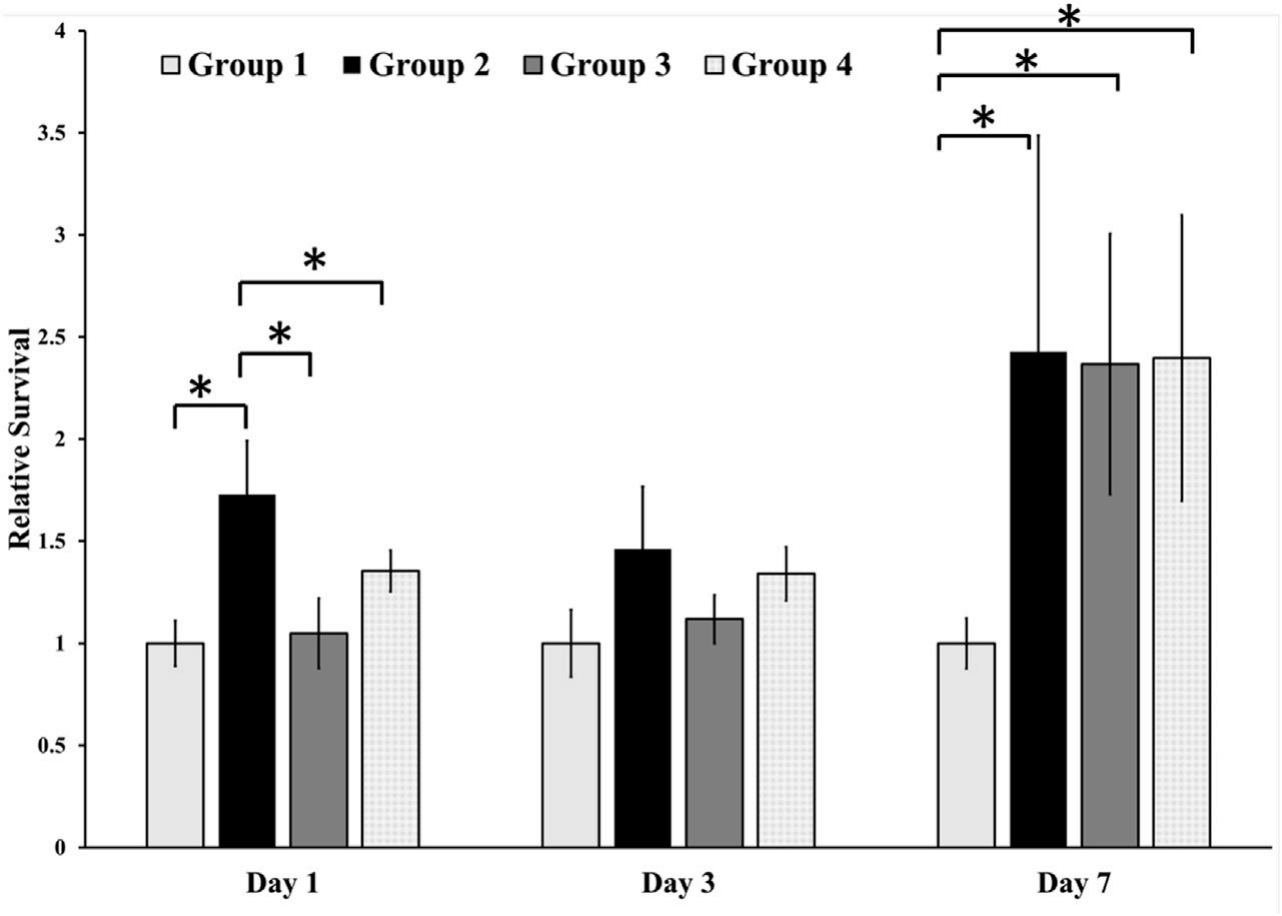
Cell toxicity of osteoblastic cells on different surfaces. We found a statistically significant difference between the relative survivability of osteoblastic cells in the treatment groups compared with the control group. Group 1—Control; Group 2—Treatment 1 (30s Sulfuric Acid +20s DI water immersion + DI Water Wash); Group 3—Treatment 2 (30s Sulfuric Acid +20s DI water immersion +30s Acetone immersion + DI Water Wash); Group 4 - Treatment 3 (30s Sulfuric Acid +20s DI water immersion +30s NaOH immersion + DI Water Wash). The “*” indicates that the difference between the groups was statistically significant (*p* < 0.05).

The cytotoxicity of the different surfaces was assessed by incubating cells on different groups of samples (Figure 4) and conducting the SRB assay at different time points. During the initial and subsequent assessments on Day 1 and Day 3, our analysis did not reveal any notable variations in relative survivability across the different groups, except for a statistically significant increase in the relative survivability of cells observed in Group 2 on Day 1. However, there was a statistically significant increase in the relative survivability of the osteoblastic cells in the treated surface on Day 7 compared with the control group. However, there was no difference found between the treated groups.

### 3.6 Alkaline phosphatase activity

**FIGURE 5:**
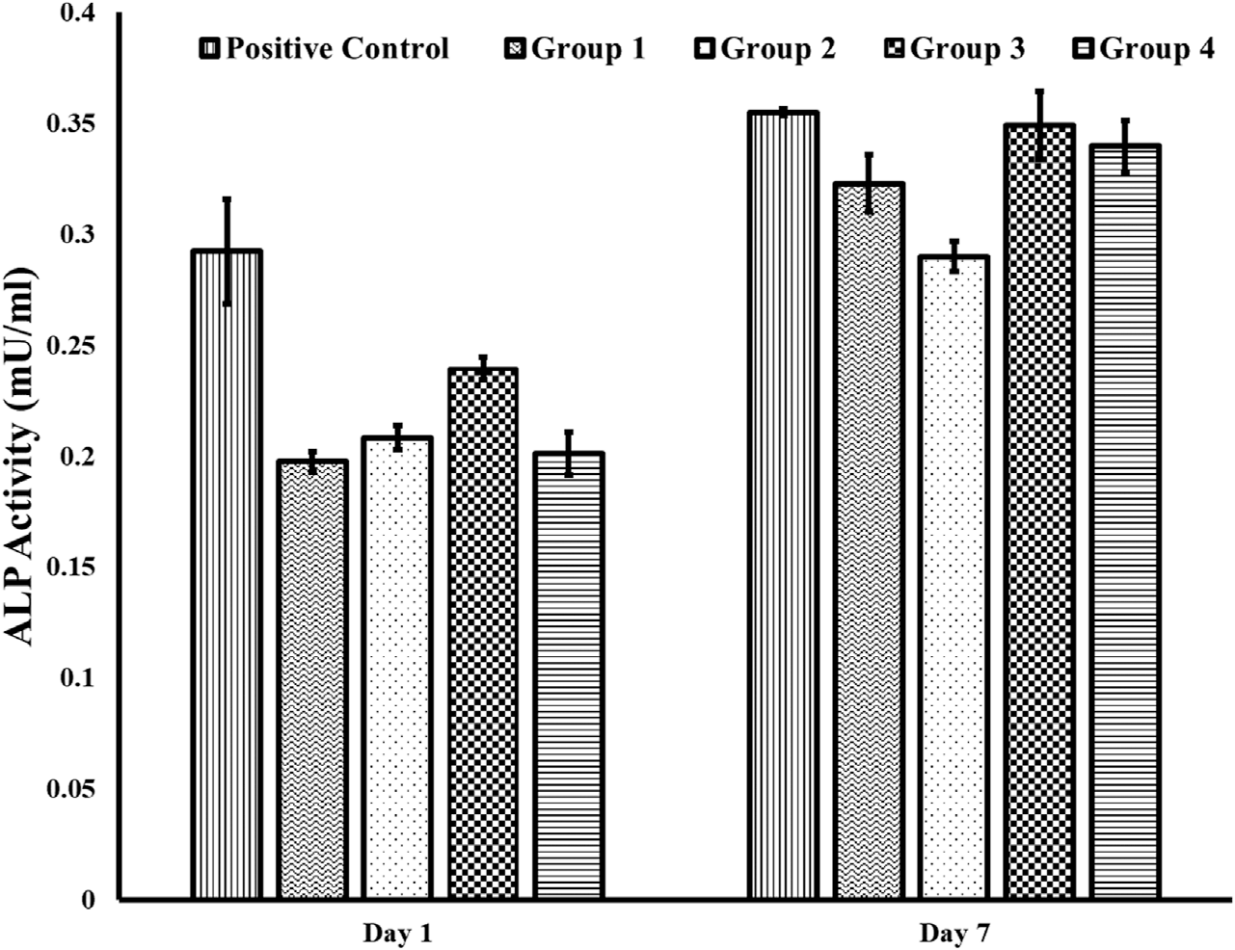
ALP activity using the fluorometric method. We found an increase in ALP activity for cells on Group 3 and 4 surfaces compared to cells on Group 2 for day 7. Further, the ALP activity on the positive control surface for day 7 was similar to the ALP activity on Groups 3 and 4. Group 1—Control; Group 2—Treatment 1 (30s Sulfuric Acid +20s DI water immersion + DI Water Wash); Group 3—Treatment 2 (30s Sulfuric Acid +20s DI water immersion +30s Acetone immersion + DI Water Wash); Group 4 - Treatment 3 (30s Sulfuric Acid +20s DI water immersion +30s NaOH immersion + DI Water Wash). The error bars indicate the standard error.

The differentiation potential of osteoblastic cells on different group surfaces was determined using an ALP assay. The results (mean standard error ± stand errors) in U/ml are summarized as follows. Over a period of 1 week, cells on Group 2 surfaces (washed only with DI water) had the lowest ALP activity of (0.29 ± 0.01 U/ml) while cells on Group 3 and Group 4 surfaces had ALP activity of 0.35 ± 0.02 U/ml and 0.34 ± 0.01 respectively. These values were similar to the activity of 0.35 ± 0.00 for cells on positive control. There was no significant difference between the ALP activity of cells on Group 3 and 4 surfaces (Figure 5).

## 4 Discussion

We found that PEEK surfaces were modified at the nano level using chemical etching with the formation of nanopores in all the treatment groups. 30 s of etching treatment with the 98 wt% concentrated sulfuric acid was sufficient for providing a porous structure ranging from 1.02 to 1.24 µm (Figure 1). We found no statistically significant difference in the pore size for different treatment groups except when compared with the control group. The resulting porous structure is due to the process of sulfonation. This sulfonation process causes the substitution of hydrogen atoms in the PEEK backbone with sulfonic acid (-SO3H) groups leading to the formation of pores/voids in the PEEK structure (Huang et al., 2001; Wang et al., 2019).

Further, these sulfonic groups introduce additional oxygen atoms, altering oxygen’s overall weight concentration, which was further confirmed by the EDX results that showed a higher weight % of oxygen in the treatment groups (Ha et al., 1997). In addition, sulfuric acid is a strong oxidizing agent and can lead to the oxidation and degradation of the polymer chains (Rimkute et al., 2022). Oxygen from the sulfuric acid molecule can react with the polymer’s carbon and hydrogen atoms, causing them to break down into smaller molecules. This can lead to a decrease in the weight concentration of carbon and hydrogen, accompanied by an increase in oxygen concentration. This might explain why the carbon weight % was decreased in the EDX results for the treatment groups. While nitrogen isn't directly involved in the reaction with sulfuric acid, the changes in the polymer’s structure due to sulfonation or oxidation can potentially affect the interaction of nitrogen-containing groups in the polymer with the surrounding environment. This might indirectly influence the apparent nitrogen concentration that was observed from the EDX analysis.

Besides sulfonation, the post-etching methods resulted in different surface chemistries on the samples, as revealed by EDX analysis (Table 1). Since the control surface was not treated with any chemical, no trace of sulfur or sodium was found on its surface. We found different weight concentrations of sulfur in the treatment groups. This may be because of the different post-processing methods. Treating with acetone would have removed sulfur (Group 3) and had a weight concentration of 0.1%, while washing with sodium hydroxide deposited sodium sulfate on the surface, thereby increasing the weight concentration of sulfur to 1.4% (Group 4). Since samples from group 4 were treated with sodium hydroxide, a weight percentage of 0.1% sodium was also found on their surface. We found different contact angles on the four other surfaces as measured from the Goniometer (Figure 2). There was a statistically significant (*p* < 0.05) increase in contact angles of the treatment groups (Group 2–4) compared with the flat polished PEEK (Group 1). The control PEEK surface had a contact angle of 71.85 ± 5.31^o^, similar to values reported previously (Wang et al., 2017; Wang et al., 2019). The treatment group’s contact angle values were increased over 90^o^ showing a shift toward the hydrophobic nature of the treated surfaces. However, because our treatment groups were porous, as revealed from the SEM images, the measured contact angle demonstrated in our study may not be a true reflection of the actual contact angle as described by the Wenzel equation, cos θ* = R cos θ where θ and θ* are respectively the measured contact angle and corrected contact angle on the rough surface, and R is the ratio of the roughened wet surface area to its projection on the apparent solid plane (Zheng and Lü, 2014). The roughness of all the treatment group samples changed from a 7.22 × 10^−3^ ± 0.001 µm for the flat polished surface (Group 1) to the range of 110–150 nm (Figure 3) for the treated groups. This roughness change is due to the formation of nanopores in the treatment groups.

The topology of the surface in the treatment groups and different surface chemistries led to different osteoblastic responses. We found that the relative survivability of osteoblastic cells on the treated surfaces increased to 2.5 times compared to the untreated PEEK. The porous structure and surface roughness of the treated groups (Groups 2–3) provided ideal conditions for osteoblastic proliferation without cell toxicity. It has been found that the rough surfaces allow osteoblastic cells to adhere and proliferate better (Matos, 2021). In addition, surface roughness at the nanoscale level can induce osteoblastic cell adhesion, growth, and differentiation (Igwe et al., 2011; Song et al., 2013), and our new surfaces were in that range. This may explain why we found a statistically significant increase in the relative survivability of the osteoblastic cells in the treated group on Day 7 compared to the untreated group. However, no statistically significant difference was found in the relative survivability of the osteoblasts based on the post-treatment of samples after etching with 98% sulfuric acid (Figure 4) since all the treated surfaces had similar pore sizes. Furthermore, up until Day 3, minimal alterations were observed in the relative cell viability across the diverse groups, which closely resembled the levels observed on Day 1. This phenomenon could be attributed to the cells’ acclimatization to the novel environment and surfaces. Following attachment, a subsequent phase of cell proliferation appears to have commenced, potentially explaining the observed pattern on Day 7.

Further, we observed an increase in ALP activity in cells in Groups 3 and 4 on Day 7, suggesting increased osteoblastic differentiation and osteogenic potential on surfaces when there was a reduced sulfur due to the sulfonated group (Group 3) or the presence of sodium sulfate (Group 4). Osteoblastic cells on Group 2 surface, where the samples were washed with water after acid etching, had a high concentration of sulfur, as indicated from the EDX analysis, and had the least ALP activity indicating that the presence of loosely attached sulfonated group does not promote osteoblastic differentiation and osteogenic potential. These results are consistent with a previous study which showed that osteoblastic cells on PEEK treated with sulfuric acid and washed with acetone, relative to osteoblastic cells on PEEK washed with water only, displayed enhanced adhesion and proliferation (Zhao et al., 2013).

In summary, our results demonstrated the surface modification of PEEK using a chemical etching method with post-processing with either acetone or sodium hydroxide at the nano level, provided a nano-porous structure with improved wettability, roughness, and surface chemistry. While there was no statistical difference in the surface properties, including surface roughness, and wettability with different post-processing chemical methods, there was a difference in the chemical composition on the surface of the PEEK in each post-processing method. And that dictated the enhanced osteoblastic cell differentiation and osteogenic potential in the group that was washed with acetone (Group 3) and sodium hydroxide (Group 4) after sulfuric acid treatment. Although in light of our findings, we suggest that surface modification of PEEK at the nanoscale level could improve osteoblastic cell differentiation and osteogenic potential with post-processing with either acetone or sodium hydroxide, it should be noted that these results are based on *in vitro* studies. Also, we were limited by our ability to calculate the corrected contact angles in this current study based on the Wenzel equation. We are currently assessing the mechanical properties of the acid-etched samples and evaluating the long-term stability of the material properties to fully understand the potential use of these modified surfaces in orthopedic and dental implant applications.

## 5 Scope of the work

The Journal of Frontiers in Bioengineering is presented with a study focused on advancing the clinical potential of Polyether-ether-ketone (PEEK) in medicine. PEEK is increasingly recognized for its mechanical robustness, dimensional stability, and chemical resistance, rendering it a promising material for medical applications. However, its innate bioinert nature hinders optimal bone osseointegration, curbing its clinical utility.

In this study, we attempted to modify the surface of PEEK at the nanoscale systematically using different post-processing chemical methods with fast ambient room temperature sulfonation using 98wt% sulfuric acid. Three distinct post-treatment methods were subsequently employed to enhance bone cell adhesion and proliferation. The experiment encompassed four PEEK sample groups: Control (Group 1), Sulfuric acid etched and distilled water washed (Group 2), Sulfuric acid etched, acetone and distilled water washed (Group 3), and Sulfuric acid etched, 4 wt% sodium hydroxide and distilled water washed (Group 4).

In-depth characterization encompassing surface topology, porosity, roughness, and wettability was conducted through sophisticated techniques, including scanning electron microscopy, profilometry, and goniometry. Energy-dispersive X-ray spectroscopy was employed for elemental analysis. Furthermore, cell toxicity and alkaline phosphatase activity (ALP) assays were conducted to gauge the biocompatibility of the materials. The results revealed enhancements in both surface morphology and cell behavior for treated groups, particularly in the case of Groups 3 and 4.

It is essential to note that the study presents a comprehensive evaluation of the proposed methodology through *in vitro* assessments. The findings substantiate the potential of surface modification through chemical etching followed by post-processing with acetone or sodium hydroxide to engender a nano-porous structure that augments osteoblastic cell differentiation and osteogenic potential, potentially paving the way for an array of enhanced clinical applications.

## Data Availability

The original contributions presented in the study are included in the article/Supplementary material, further inquiries can be directed to the corresponding author.
